# Age Does Not Affect Respiratory Characteristics in Children With Prader‐Willi Syndrome Before and After Growth Hormone Therapy

**DOI:** 10.1111/apa.70459

**Published:** 2026-01-23

**Authors:** Michelle Ip, Sze Lut Cheng, Okkes R. Patoglu, Georgina Plunkett, Lauren C. Nisbet, Gillian M. Nixon, Margot J. Davey, Rosemary S. C. Horne

**Affiliations:** ^1^ Department of Paediatrics Monash University Melbourne Australia; ^2^ Melbourne Children's Sleep Centre Monash Children's Hospital Melbourne Australia

**Keywords:** central sleep apnoea, growth hormone, obstructive sleep apnoea, Prader‐Willi syndrome, sleep

## Abstract

**Aim:**

Children with Prader‐Willi syndrome (PWS) are at increased risk of both central (CSA) and obstructive sleep apnoea (OSA). Studies examining the effects of growth hormone have focused on older children; however, therapy is often initiated before the age of 2 years. We determined the effects of age on (1) the number of children diagnosed with OSA and CSA; (2) the sleep and respiratory characteristics and (3) the effects of growth hormone on OSA and CSA.

**Methods:**

Retrospective review of children with PWS who underwent polysomnography pre‐ and post‐growth hormone between January 2011 and June 2024.

**Results:**

Fifty‐six children (35 < 2 years; 21 ≥ 2 years) pre‐growth hormone; 28 children < 2 years and 15 children ≥ 2 years after growth hormone. Pre‐growth hormone, children ≥ 2 years had more severe OSA than children < 2 years (*p* < 0.05). There was no difference between age groups for CSA. Post‐growth hormone, 21% of children < 2 years and 20% of children ≥ 2 years developed OSA. CSA resolved post‐growth hormone in 21% of children < 2 years and 6% of children ≥ 2 years, whilst CSA developed in 11% and 13%, respectively.

**Conclusion:**

Our study highlights that very young children do not appear to be at higher risk of development of OSA or CSA when treated with growth hormone.

## Summary

- Previous studies examining the effects of growth hormone on sleep apnoea have been in older children with Prader‐Willi syndrome.
- We did not identify any effects of age on the development of obstructive and central sleep apnoea after growth hormone initiation.
- Our study highlights that children under 2 years of age do not appear to be at higher risk of development of obstructive and central sleep apnoea after growth hormone initiation.

**Abbreviations**
ANOVA: analysis of variance
BMI: body mass index
CAHI: central apnoea–hypopnoea index
CSA: central sleep apnoea
IQR: interquartile range
NREM: non‐rapid eye movement sleep
OAHI: obstructive apnoea–hypopnoea index
OSA: obstructive sleep apnoea
PWS: Prader‐Willi syndrome
RDI: respiratory disturbance index
REM: rapid eye movement sleep
SpO_2_
: peripheral oxygen saturation
TcCO_2_
: transcutaneous carbon dioxide
TST: total sleep time
WASO: wake after sleep onset

## 1 Introduction

Individuals with Prader‐Willi syndrome (PWS) have an increased risk of sleep‐disordered breathing (SDB) including obstructive sleep apnoea (OSA), central sleep apnoea (CSA) and reduced ventilatory and arousal responses to hypoxic and hypercapnic stimuli during sleep [1]. Growth hormone is now being prescribed for infants and young children with PWS following reports of the benefits of early growth hormone treatment on growth and body composition, motor function and neurocognitive development [2]. However, there have been reports of children who have died in the early phases of growth hormone treatment, with the majority of these being during sleep [3]. Although no causal association between growth hormone treatment and sudden death in individuals with PWS has been identified, several of the children had symptoms of OSA prior to their sudden death during sleep, raising concern about the possibility of growth hormone contributing to the risk of sudden death due to either development of upper airway obstruction or worsening pre‐existing OSA.

In Australia, growth hormone has been licensed for children with PWS since 2009. Australian guidelines for government funding for initiation of growth hormone for the treatment of PWS require that the child has been evaluated for airway obstruction and apnoea, including having had a comprehensive polysomnographic study within the previous 12 months, and that any sleep disorder requiring treatment has been addressed. A previous cross‐sectional study of children aged 3 months–16.3 years with PWS referred for sleep evaluation to the Melbourne Children's Sleep Centre prior to commencing growth hormone between 2003 and 2011 identified that 44% had OSA and 12% had CSA [4]. Those diagnosed with OSA were older than those without OSA (9.8 ± 4.6 vs. 5.3 ± 4.8 years, *p* = 0.009). Importantly, clinical symptoms of SDB, including breathing problems during sleep reported by parents, enlarged tonsils and body mass index (BMI) did not distinguish between those with and without OSA [4]. According to Expert Consensus Guidelines, there is strong evidence supporting the use of growth hormone for PWS beginning in infancy, with over 70% of children now commencing growth hormone before 2 years of age [5]. Commencing growth hormone in the first year of life compared to 2–5 years of age has been shown to be safe and had more beneficial effects on body composition after 8 years [6]. Children under 2 years are more likely to have CSA [7] and our previous studies have shown that the effects of centrally mediated disturbance in breathing on heart rate, blood pressure and cerebral oxygenation are similar to those of obstructive respiratory events [8]. The impact of growth hormone on CSA in this group of young children has not been described. In addition, previous studies of the development of OSA on growth hormone are based on older cohorts of children where increased growth of adenoid and tonsillar tissue may contribute to the development of OSA [9] and the findings may therefore not apply to the very young cohort currently being prescribed growth hormone therapy. Information about the impact of growth hormone on OSA and CSA in infants and young children will help guide clinical decision‐making around the time of growth hormone commencement.

The aim of this retrospective review of children with PWS was to determine the effects of age on (1) the number of children who were diagnosed with OSA and CSA prior to receiving growth hormone; (2) the sleep and respiratory characteristics of these children and (3) the effects of growth hormone on OSA and CSA after commencement of treatment. We hypothesised that CSA would be more common and OSA less common in children under 2 years of age, and that these might worsen after treatment with growth hormone.

## 2 Methods

This study received approval from the Monash Health Human Research Ethics Committee (RES‐24‐0000‐503Q) as a clinical audit project which did not require consent for inclusion. A search of the sleep laboratory database identified 56 children with PWS who had a pre‐growth hormone screening polysomnographic study between 01/01/2011 and 23/06/2024. Recommendations for treatment following the baseline polysomnographic study were collected. If multiple polysomnographic studies were performed, the study closest to growth hormone initiation and the first study within a year of commencing growth hormone were selected for analysis.

All polysomnographic studies were conducted in accordance with the paediatric guidelines of the American Academy of Sleep Medicine current at the time of the study [10, 11, 12]. Prior to the polysomnographic studies, height and weight were measured and body mass index (BMI) *z*‐score calculated. Obesity was defined as ≥ 95th percentile (BMI *z*‐scores ≥ 1.65) and overweight as ≥ 85th percentile (BMI *z*‐scores ≥ 1.04) [13]. Electrophysiological signals were recorded using a commercially available polysomnographic system (E‐Series or Grael, Compumedics, Melbourne, Australia) and included electroencephalogram (Cz, F3‐M2, F4‐M1, C3‐M2, C4‐M1, O1‐M2 and O2‐M1), right and left electrooculogram, submental electromyogram, left and right anterior tibialis electromyogram, and electrocardiogram. Respiratory characteristics were captured using abdominal and thoracic respiratory plethysmography (Pro‐Tech zRIP Effort Sensor; Pro‐Tech Services Inc., Mukilteo, WA), oronasal thermistor, nasal pressure and transcutaneous carbon dioxide (TcCO_2_) (TCM4/40; Radiometer, Denmark, Copenhagen or Sentec, Therwil, Switzerland). Peripheral oxygen saturation (SpO_2_) was measured using Bitmos GmbH (Bitmos, Dusseldorf, Germany), which uses Masimo signal extraction technology for signal processing, or Masimo Radical 7 (Masimo, Irving, CA), with both devices set to a 2‐s averaging time.

### 2.1 Sleep and Respiratory Analysis

All polysomnographic studies were scored manually by trained paediatric sleep scientists using Compumedics ProFusion software. For studies recorded prior to 2012, NREM 3 and NREM 4 were combined into N3 to be consistent with current staging rules. For infants younger than 3 months of age, sleep was scored as active sleep and quiet sleep. Total sleep time (TST) was defined as the total time spent asleep, sleep latency as the time from lights out to the first epoch of sleep, and sleep efficiency as the percentage of TST given the time available for sleep. Wake after sleep onset (WASO) was defined as the time spent awake during the sleep period, and the arousal index as the total number of arousal events per hour of sleep. Scoring of respiratory events (> 2 breaths in duration) was as follows: An obstructive apnoea was defined as the cessation of airflow in association with ongoing respiratory effort; an obstructive hypopnoea was defined as ≥ 30% decrease in nasal pressure signal amplitude, associated with increased work of breathing and an arousal or ≥ 3% decrease in oxygen saturation; a central apnoea was defined as cessation of airflow without inspiratory effort lasting either ≥ 20 s or at least the duration of two breaths and associated with an arousal or ≥ 3% oxygen desaturation; and a central hypopnoea as ≥ 30% decrease in nasal pressure signal amplitude with reduced inspiratory effort throughout the entire duration of the event and associated with an arousal or ≥ 3% oxygen desaturation. A mixed apnoea was defined if an event was associated with absent respiratory effort during one portion of the event and the presence of obstructed inspiratory efforts in another portion, regardless of which portion came first. The obstructive apnoea–hypopnea index (OAHI), defined as the total number of obstructive and mixed apnoeas and hypopneas per hour of TST, was used to define OSA severity. Primary snoring was defined as an OAHI ≤ 1 event/h, mild OSA as an OAHI of > 1 ≤ 5 events/h, and moderate/severe OSA as an OAHI of > 5 events/h. In addition, the central apnoea–hypopnea index (CAHI), defined as the total number of central apnoeas and hypopneas per hour of TST, was used to define the presence of CSA (> 5 events/h). The respiratory disturbance index (RDI) was defined as the total number of obstructive, central and mixed apnoeas and hypopneas per hour of sleep. SpO_2_ nadir was the lowest oxygen saturation following a respiratory event, and the average SpO_2_ drop was the average SpO_2_ drop that occurred following a respiratory event.

### 2.2 Statistical Analysis

All statistical analyses were performed with Sigma Plot (Systat Software Inc. Version 14.5). Data were first tested for normality and equal variance. Demographic, sleep and respiratory data were compared with Student's *t*‐tests if normally distributed and Mann Whitney *U*‐tests if not. Percentages of children in each sleep‐disordered breathing category were compared with a Chi‐Square test, or a Fisher Exact test if any cell contained < 5 observations. Respiratory data pre‐ and post‐growth hormone initiation were compared between the two age groups with 2‐way analysis of variance (ANOVA), with post hoc Bonferroni tests if differences were identified. As the majority of data were non‐parametric, data are presented as median and interquartile range (IQR). Statistical significance was taken at *p* < 0.05.

## 3 Results

We identified 56 children who had a pre‐growth hormone study between 1 January 2011 and 23 June 2024. Children (26 girls) ranged in age from 2 months to 19 years, with a median age of 13.5 months (IQR 9.3–42.8 months). Height was recorded in 37 and weight in 44 children.

Children were divided into two groups, those under 2 years of age (*n* = 35) and those over 2 years of age (*n* = 21). Demographic data by age group are presented in Table 1. Polysomnographic studies in the children under 2 years were evenly split between the first half of the study period (2011–2017: 19/35, 54%) and the second half (2018–2024: 16/35, 46%). In contrast, most children in the ≥ 2 age group had their first polysomnographic study in the period from 2011 to 2017 (19/21, 90%, *p* = 0.005), reflecting a change in clinic practice towards earlier commencement of growth hormone.

**TABLE 1:** Comparison of demographics at baseline between children with Prader‐Willi syndrome younger than 2 years of age and older than or equal to 2 years of age.

	< 2 years of age (*n* = 35)	≥ 2 years of age (*n* = 21)
Sex	15 girls/18 boys	10 girls/11 boys
Age at study (years)	0.9 (0.5–1.1)	6.6 (3.0–9.9)***
Height (cm)	66 (63–73) [*n* = 18]	121 (85–132)*** [*n* = 19]
Weight (kg)	7.9 (6.4–8.8) [*n* = 24]	33.2 (11.2–53.3)*** [*n* = 20]
BMI (kg/m^2^)	16.6 (15.9–18.0) [*n* = 18]	23.9 (17.3–29.4)*** [*n* = 19]
BMI *z*‐score		1.9 (0.7–2.6) [*n* = 18]
% Obese		56%

*Note:* Values are median (IQR). *p < 0.05, **p < 0.01, ***p < 0.001 compared between the two age groups of children. BMI *z*‐scores were not available for children under the age of two.

### 3.1 Pre‐Growth Hormone Sleep Study Results

Table 2 shows the comparison of sleep and respiratory data between the two age groups. Sleep was scored as active sleep or quiet sleep in 3 children < 3 months of age and their data are only included in %NREM and %REM sleep. The children younger than 2 years of age had a shorter REM sleep latency (*p* < 0.01), lower %N2 (*p* < 0.05) and %NREM (*p* < 0.01) and higher %REM sleep (*p* < 0.001), as expected.

**TABLE 2:** Comparison of polysomnographic sleep and respiratory variables at baseline between children with Prader‐Willi syndrome younger than 2 years of age and older than or equal to 2 years of age.

	< 2 years of age (*n* = 35)	≥ 2 years of age (*n* = 21)
Sleep latency (min)	11 (4–28)	20 (6–36)
Sleep period time (min)	513 (496–552)	505 (453–536)
Total sleep time (min)	463 (436–486)	456 (400–477)
Sleep efficiency (%)	86 (82–89)	86 (79–90)
REM latency (min)	48 (36–60)**	82 (62–126)
WASO (SPT)%	10 (8–13)	10 (6–14)
N1%	8 (4–10) [*n* = 31]	7 (7–12)
N2%	41 (34–48)* [*n* = 32]	46 (38–49)
N3%	25 (20–29) [*n* = 32]	23 (16–26)
NREM%	72 (67–77)**	78 (73–82)
REM%	28 (23–33)***	22 (17–25)
No OSA (*n*, %)	30 (85.7)	13 (61.9)
Mild OSA (*n*, %)	3 (8.5)	3 (14.2)
Moderate/severe OSA (*n*, %)	2 (5.7)	5 (23.8)
No CSA (*n*, %)	23 (65.7)	15 (71.4)
CSA > 5 events/h (*n*, %)	12 (34.3)	6 (28.6)
RDI (events/h)	4.7 (2.9–11)	5.6 (3.8–9.4)
No OSA (*n*, %)	30 (85.7)	13 (61.9)
Mild OSA (*n*, %)	3 (8.5)	3 (14.2)
Moderate/severe OSA (*n*, %)	2 (5.7)	5 (23.8)
No CSA (*n*, %)	23 (65.7)	15 (71.4)
CSA > 5 events/h (*n*, %)	12 (34.3)	6 (28.6)
RDI (events/h)	4.7 (2.9–11)	5.6 (3.8–9.4)
REM RDI (events/h)	10 (5–18)	12 (8–27)
SpO_2_ nadir (%)	85 (76–89)	84 (74–88)
Avg. SpO_2_ drop (%)	5 (4–6)	5 (4–5)
SpO_2_ < 90% (events/h)	0.5 (0.1–2.1)	0.5 (0.1–1.1)
SpO_2_ ≥ 4% (events/h)	3 (1–6)	3 (1.5–4)
Arousal index (/h)	9 (6–11)	9 (8–13)
Average TcCO_2_ TST (mmHg)	40 (37–44)***	46 (43–48)

*Note:* Values are median (IQR) or number and (%). *p < 0.05, **p < 0.01, ***p < 0.001 compared between the two age groups of children.

Abbreviations: Avg. SpO_2_ drop, average SpO_2_ desaturation drops; CSA, central sleep apnoea; OSA, obstructive sleep apnoea; RDI, respiratory disturbance index; SpO_2_ < 90%, SpO_2_ desaturation index to < 90%; SpO_2_ ≥ 4%, SpO_2_ desaturation index with desaturation ≥ 4%; TcCO_2_, transcutaneous carbon dioxide; TST, total sleep time; WASO, wake after sleep onset.

**FIGURE 1:**
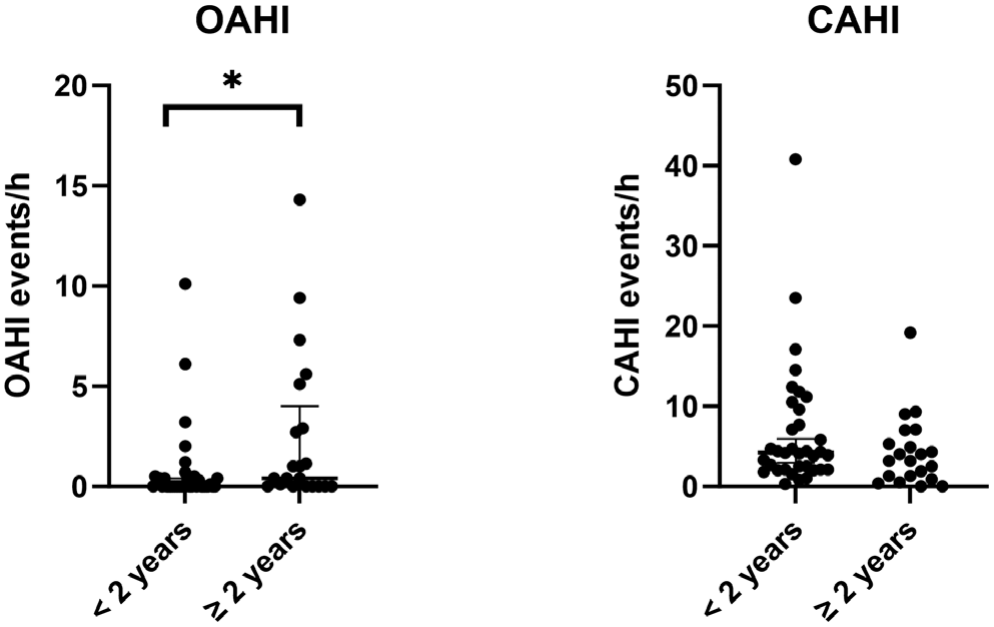
Comparison of obstructive apnoea–hypopnoea index (OAHI) and central apnoea–hypopnoea index (CAHI) in children under 2 years of age and 2 years and over at their pre‐growth hormone polysomnographic study. Values are median and IQR. *p < 0.05.

OAHI and CAHI prior to growth hormone therapy are compared between the two age groups in Figure 1. Children with PWS ≥ 2 years of age had a significantly higher OAHI (*p* = 0.032) than children < 2 years of age. There was a trend for more children in the < 2 years age group to not have OSA (85.7%) compared to the older children (61.9%, *p* = 0.054), with no difference between the groups for the number of children with mild OSA and a trend for more children in the older group to have moderate/severe OSA (*p* = 0.09) (Table 2). In the children ≥ 2 years of age 9/10 who were obese had OSA. In the children < 2 years of age none had both CSA and OSA and in the children ≥ 2 years 2 children (10%) had both. There was no difference between groups for CAHI or for the percentage of children who had CSA (34.3% in the children < 2 years and 28.6% in the children ≥ 2 years). The only other difference between the age groups was the average TcCO_2_ levels were significantly higher in children ≥ 2 years of age (*p* < 0.001).

**FIGURE 2:**
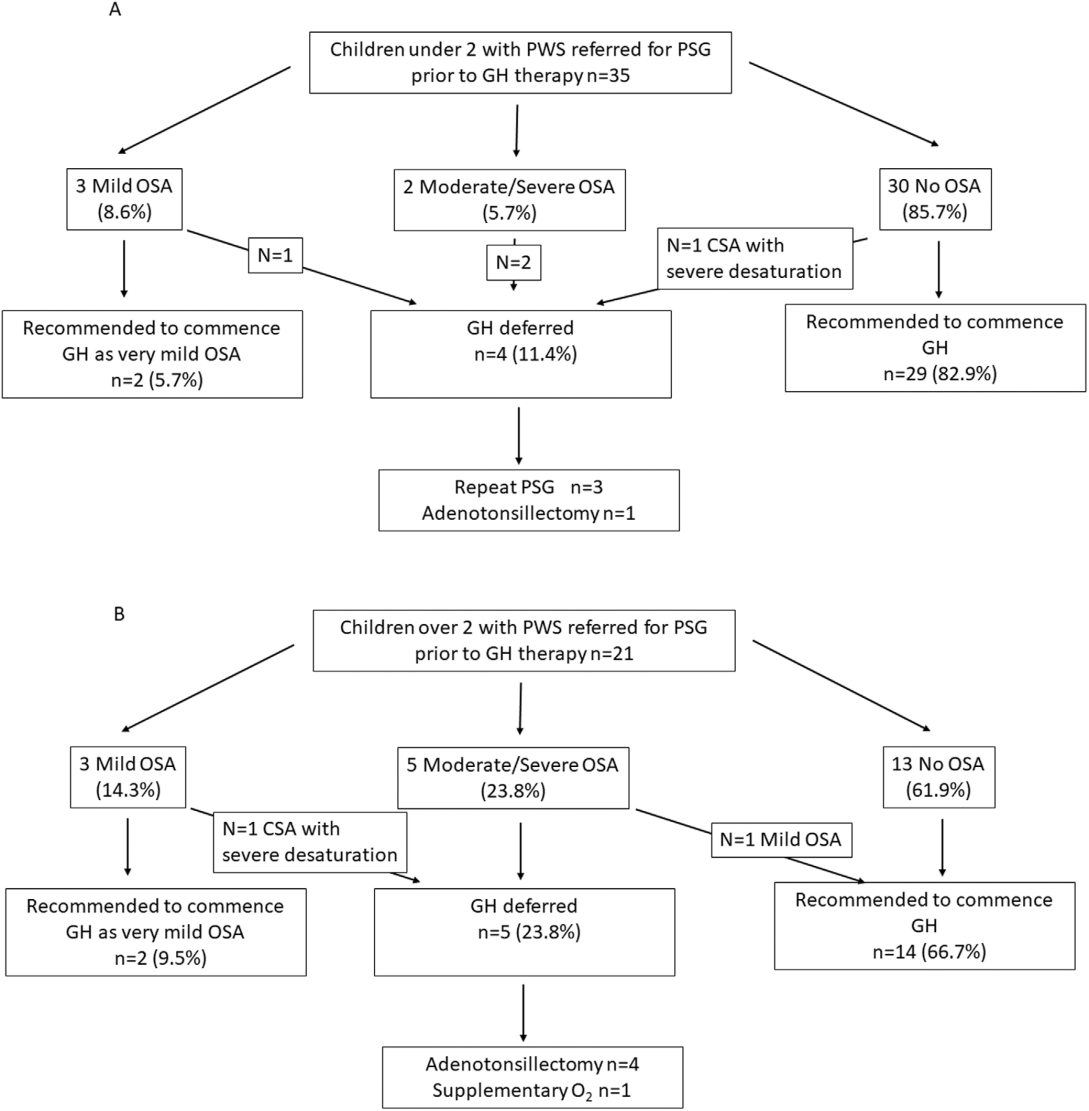
(A) Clinical recommendations for children under 2 years of age following polysomnography prior to growth hormone therapy. (B) Clinical recommendations for children ≥ 2 years of age following polysomnography prior to growth hormone therapy. CSA, central sleep apnoea; OSA obstructive sleep apnoea.

The treatment recommended based on the polysomnographic study for the children in the two age groups is presented for those under 2 years (Figure 2A) and over 2 years (Figure 2B). Commencement of growth hormone was recommended in 31/35 (89%) of children under 2 years and 15/21 (71%) of children over 2 years (*p* = 0.15), mainly in those without OSA. One child in each age group with no OSA but CSA associated with significant desaturations was recommended to defer growth hormone commencement. Two of the 3 children with extremely mild OSA in each group were recommended to commence growth hormone therapy, with one child < 2 years recommended to have a repeat polysomnographic study in 6 months and one ≥ 2 years recommended to have treatment of concurrent CSA prior to consideration of growth hormone. Growth hormone therapy was deferred for all the children with moderate/severe OSA while they undertook treatment of OSA, apart from one child > 2 years with an OAHI of 5.1 events/h due to a cluster of obstructive events at the beginning of the night associated with a period of sleep instability that was not reflected in evidence of obstruction later in the night.

### 3.2 Follow‐Up After Commencement of Growth Hormone

Post‐growth hormone polysomnographic data were available for 28 children who were < 2 years at the pre‐growth hormone polysomnographic study and 15 children who were ≥ 2 years, as some children were lost to follow‐up or had not been recommended for growth hormone treatment. Time between studies was affected by external factors such as clinic scheduling and the start date for growth hormone prescribing, noting that the post‐growth hormone study is recommended 6–8 weeks after commencement of growth hormone.

Demographic and respiratory data pre‐ and post‐growth hormone in the two age groups are presented in Table 3. In the < 2 group the median age for the pre‐ growth hormone study was 10 months (range: 5.0–13.8 months) and the median age at the post‐growth hormone study was 16.2 months (range: 10.0–21.5 months) with the median time between studies of 5.5 months (range: 4.0–7.0 months). In the ≥ 2 group the median age at the pre‐ growth hormone study was 70 months (range: 28–109 months) and the median age at the post‐growth hormone study was 80 months (range: 37–118 months) with a median time between studies of 8 months (range: 6.0–10.0 months). There was no change in BMI *z*‐score between studies in either age group. Average TcCO_2_ levels were significantly higher in children ≥ 2 years of age compared to those < 2 years during both the baseline and post‐growth hormone studies (*p* < 0.01 for both).

**TABLE 3:** Demographic and respiratory characteristics of the children under 2 and 2 years and over pre‐ and post‐growth hormone therapy commencement.

	< 2 years of age (*n* = 28)		≥ 2 years of age (*n* = 15)	
	Pre‐growth hormone	Post‐growth hormone	Pre‐growth hormone	Post‐growth hormone
Age (months)	10.0 (5.0–13.8)***	16 (10.0–23.5)	70.0 (28.0–109.0)***	80.0 (37.0–118.0)
Height (cm)	64.5 (57.9–74.5)***	71.5 (62.3–82.5)	112.3 (84.0–134.8)***	111.5 (85.5–137.5)
Weight (kg)	7.5 (5.2–8.3)***	9.6 (7.8–10.2)	23.2 (10.6–49.8)***	24.5 (13.7–52.5)
BMI	16.2 (15.0–17.8)	17.1 (16.1–18.7)	21.1 (17.1–27.0)	25.1 (17.4–28.2)
BMI *z*‐score	—	—	1.43 (0.69–2.41)	1.41 (1.02–2.33)
RDI (events/h)	4.5 (2.5–11.2)	4.6 (2.1–8.1)	5.0 (4.0–8.3)	5.7 (4.6–7.6)
OAHI (events/h)	0.0 (0.0–0.4)	0.2 (0.0–0.7)	0.4 (0.0–1.0)	0.7 (0.0–1.6)
Mild OSA (*n*, %)	1 (4%)	4 (14%)	3 (20%)	6 (40%)
Moderate/severe OSA (*n*, %)	0	2 (7%)	0	0
CAHI (events/h)	4.3 (2.1–11.0)	3.8 (1.8–5.7)	4.3 (1.3–7.1)	5.0 (2.8–6.0)
CSA > 5 events/h (%)	11 (39%)	7 (25%)	6 (40%)	7 (47%)
REM RDI (events/h)	8.0 (4.2–17.7)	9.1 (5.2–14.1)	11.1 (7.2–24.3)	12.2 (8.6–25.4)
SpO_2_ nadir (%)	85.0 (79.3–89.8)	84.0 (80.5–89.0)	84.0 (74.0–89.0)	81.0 (73.0–88.0)
Avg. SpO_2_ drop (%)	5.0 (4.0–5.0)	5.0 (4.0–6.0)	4.0 (4.0–5.0)	5.0 (5.0–6.0)
SpO_2_ < 90% (events/h)	0.5 (0.0–1.6)	0.7 (0.1–1.3)	0.5 (0.1–1.0)	1.1 (0.1–2.1)
SpO_2_ ≥ 4% (events/h)	2.8 (1.0–6.7)	2.8 (1.1–5.1)	3.1 (1.4–4.0)	3.4 (1.7–5.2)
Arousal index (/h)	8.4 (6.2–11.8)	8.4 (6.5–11.8)	8.7 (6.7–12.7)	9.3 (5.9–11.7)
Average TcCO_2_ TST (mmHg)	41.7 (38.1–43.7)**	42.0 (38.5–45.1)**	46.1 (42.5–48.2)	46.5 (41.2–50.5)

*Note:* Values are median and IQR. **p < 0.01; ***p < 0.001 between pre and post growth hormone studies.

Abbreviations: Avg. SpO_2_ drop, average SpO_2_ desaturation drops; CAHI, central apnoea hypopnoea index; CSA, central sleep apnoea; OAHI, obstructive apnoea hypopnoea index; RDI, respiratory disturbance index; SpO_2_ < 90%, SpO_2_ desaturation index to < 90%; SpO_2_ ≥ 4%, SpO_2_ desaturation index with desaturation ≥ 4%; TcCO_2_, transcutaneous carbon dioxide; TST, total sleep time.

**FIGURE 3:**
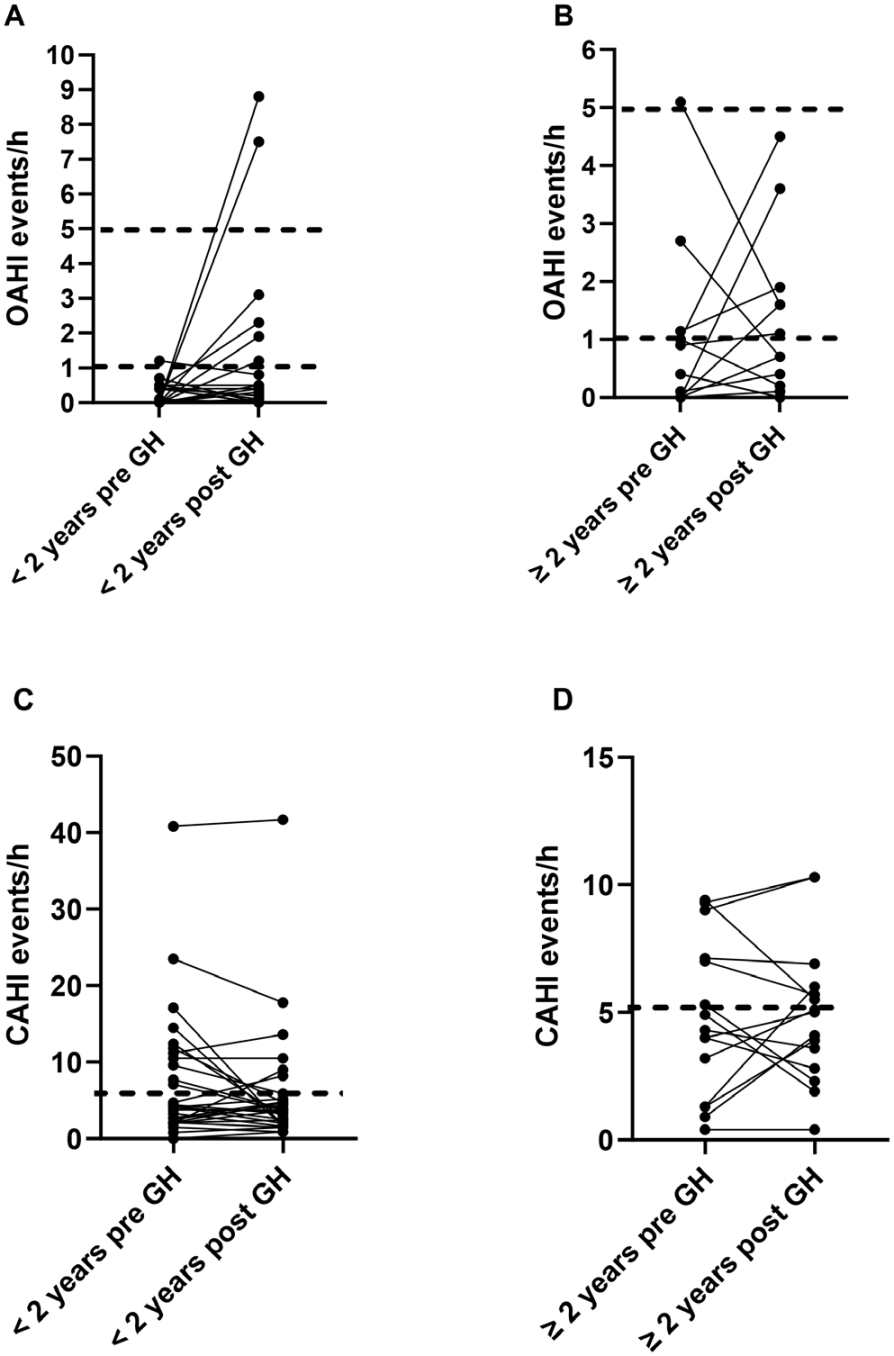
Obstructive apnoea hypopnoea index (OAHI): (A) in children under 2 years of age; (B) in children 2 years and over of age; Dotted lines at 1 and 5 events/h indicate the upper and lower cut offs for mild obstructive sleep apnoea. Central apnoea hypopnoea index (CAHI): (C) in children under 2 years of age; (D) in children over 2 years of age before and after growth hormone treatment. The dotted line at 5 events/h indicates the clinical cut off for central sleep apnoea in children.

Changes in OAHI between the pre‐ and post‐growth hormone studies are presented in Figure 3. In the < 2 group, 6 children (21%) developed OSA: 4 (14%) mild OSA and 2 (7%) moderate OSA (Figure 3A). In the ≥ 2 group, 3 children developed mild OSA (20%) and in 2 children with mild OSA at baseline the OAHI was reduced (Figure 3B). The changes in CAHI between studies are also presented in Figure 3. In the children < 2 years, 6 children (21%) no longer had CSA on the post‐growth hormone polysomnographic study, 4 children (14%) continued to have CSA and 3 children (11%) developed CSA (Figure 3C). In the ≥ 2 group, 1 child no longer had CSA (6%), 5 children continued to have CSA (33%) and 2 children developed CSA (13%) (Figure 3D). There was no difference between groups for the % of children with resolved CSA after growth hormone (*p* = 0.39).

## 4 Discussion

Children with PWS are now recommended to commence growth hormone therapy early, with the majority commencing treatment before 2 years of age [14]. Previous studies have demonstrated development of OSA in a subset of children after starting growth hormone but have focused on children over 2 years. This was the first study to specifically examine the effects of growth hormone therapy on sleep and both obstructive and central respiratory patterns in children under 2 years of age compared to older children. We identified that older children had a higher average OAHI and were more likely to have OSA before starting growth hormone. In contrast to our hypothesis, the prevalence of CSA was similar between the groups, although the mean CAHI tended to be higher in the younger children. Following growth hormone initiation, 20% of children in both groups developed OSA, whereas 21% of the children < 2 and 6% ≥ 2 had resolution of CSA. Our study highlights that despite differences in OSA severity between the age groups, initiation of growth hormone treatment did not have differing effects on the development of sleep‐disordered breathing.

In this cohort of children, the older group were more likely to have OSA (38%) compared to the children < 2 years of age (14%), and more likely to have moderate–severe OSA (23.8% compared to 5.7%) as has been reported previously [4]. Previous studies from our sleep laboratory in a smaller cohort (*n* = 17) of older children with PWS (aged 6–18 years) using the same definitions of OSA and CSA as the current study identified a similar prevalence of CSA and OSA (18% CSA, 24% OSA and 29% both) [15]. A study with a similar design to the current study also showed a higher prevalence of OSA in older children ≥ 2 years compared to < 2 years (17% vs. 48%, *p* = 0.032) [7]. Differences between studies are likely attributable to slightly different thresholds for defining OSA (1.5 events/h) or a higher percentage of the older children being obese (81% vs. 56%) [7]. Higher rates of obesity in older children with PWS likely contribute to the higher prevalence of OSA [16]. The higher prevalence of OSA in the older children is likely also due to adenotonsillar hypertrophy, which peaks between the ages of two and eight, when the most marked increase in adenotonsillar size relative to airway diameter occurs [17].

In contrast to our hypothesis we did not identify any differences between our age groups for CAHI or the prevalence of CSA. This is in contrast to the study by Cohen et al., which identified a significantly higher prevalence of CSA (43%) in the children < 2 compared to 5% in the children > 2 group. The authors used the same criteria for CSA as in our study (> 5 events/h). It is unclear why the incidence of CSA was so low in the older children in the Cohen et al., study. In a multicentre Australian study which included 94 children studied prior to growth hormone treatment (aged 0.1–13.2 years) 19% of the children had CSA [18], and a similar prevalence has been reported in other studies: 22.6% reported in children aged 6.9 ± 4.4 years [19] and 24% in children aged 1.1–17.2 years, median 7.7 years [20]. It is thought that the increased incidence of CSA in children with PWS compared to typically developing children is due to hypotonia, brainstem immaturity and hypothalamic dysfunction, together with abnormal sensitivity to CO_2_ which are features of the condition [21].

Following initiation of growth hormone treatment, 21% of the children under 2 years and 20% of the children over 2 years developed OSA. Studies have consistently reported that a subset of children with PWS develop OSA after starting growth hormone, albeit at slightly differing rates, with the lack of precision in the estimate likely due to the relatively small cohorts in each study of this rare condition. In a large multi‐centre study of 94 children, 13% of children with no or mild OSA developed moderate/severe OSA after starting growth hormone and the changes were similar for children older and younger than 3 years of age [18]. In a study of 62 children with PWS aged 0–2.5 years of whom 21 started growth hormone therapy during the first year of life and 41 after 1 year of age there were no differences in OAHI or CAHI between the groups after 3 months of growth hormone, but the incidence of OSA increased [22]. In a study of 25 patients aged 0.7–34 years who were asked to stop growth hormone for 3 months and have a baseline sleep study before restarting growth hormone therapy with another sleep study after 6 weeks, 6 patients had a worsening of AHI [23]. In a longitudinal study of 15 children aged 0.8–15 years 2 children developed severe OSA (13%) and growth hormone therapy was discontinued [24]. The remaining children were followed up at 6 months, 1 and 2 years post‐growth hormone with no significant group changes in OAHI or CAHI [24]. In another longitudinal study of 50 children aged 0.4–7.8 years, the percentage of patients with OSA increased from 3% before growth hormone initiation to 22% at 6 weeks, 21% at 6 months, 21% at 12 months, 36% at 24 months, 33% at 36 months and 38% at 48 months [9]. The increase in OAHI was directly correlated with adenoid size and severe tonsillar hypertrophy was more common at the later studies [9]. Another study found that worsening SDB while on growth hormone was associated with higher BMI, lower growth hormone dose, higher IGF‐1 levels and a non‐15q deletion [25]. In our study there was no change in BMI or BMI *z*‐score in either age group of children after growth hormone initiation to explain the development of OSA. The finding that OSA can develop on growth hormone supports the continued ongoing clinical surveillance of these children.

We also did not identify any difference in mean CAHI or the percentage of children with CSA in either age group after growth hormone initiation. We would have expected CAHI to decrease particularly in the children under 2 years of age as central apnoeas are much more common in young children and decrease with age as respiratory control improves [26]. Previous studies have shown that CSA improves with age in children with PWS [9, 27] while others have shown no change [28], with this difference potentially being related to age. The higher CO_2_ seen in our older cohort is consistent with a previous report of CSA contributing to sleep‐related hypoventilation in older children with PWS [15] and a report of more hypoventilation in children with PWS compared to typically developing children matched for OSA severity [29], reflecting reduced responsiveness to CO_2_ [1].

Consistent with many other studies, our study was a single centre study and given the rarity of PWS our sample size was small. In addition, some children were receiving care for PWS at another centre and were lost to follow‐up. We did not have access to any growth hormone‐IGF‐I signalling measurements or the dose of growth hormone administered in the children. Although there was no difference in BMI or BMI *z*‐score between studies, we unfortunately did not have measurements of neck, waist, and hip, which may have provided more information on body composition changes after growth hormone initiation. We also acknowledge that we did not have a control group of children who had not received growth hormone.

In conclusion, we identified that prior to growth hormone treatment OSA was more common in children ≥ 2 years compared to < 2 years of age; however, there was no difference in the prevalence of CSA. After growth hormone initiation, OSA developed in 20% of children in both age groups, whereas there was no difference in the % of children with resolved CSA. Our study highlights that despite differences in OSA severity at baseline between the age groups, initiation of growth hormone treatment did not have any differing effects. Importantly, we found that very young children do not appear to be at higher risk of developing OSA than older counterparts, which is reassuring clinically given the younger age of starting growth hormone in recent years.

## Author Contributions

**Michelle Ip:** investigation, formal analysis, writing – review and editing. **Sze Lut Cheng:** investigation, formal analysis, writing – review and editing. **Okkes R. Patoglu:** investigation, writing – review and editing. **Georgina Plunkett:** investigation, writing – review and editing. **Lauren C. Nisbet:** writing – review and editing. **Gillian M. Nixon:** writing – review and editing. **Margot J. Davey:** writing – review and editing. **Rosemary S. C. Horne:** concetualisation, supervision, writing – original draft preparation.

## Funding

Mr. Okkes Patoglu is supported by a Monash University graduate scholarship. Prof Rosemary Horne is supported by a National Health and Medical Research Council of Australia Investigator Grant (1195453).

## Conflicts of Interest

The authors declare no conflicts of interest.

## Data Availability

The data that support the findings of this study are available on request from the corresponding author. The data are not publicly available due to privacy or ethical restrictions.
